# Good, bad, different or something else? A scoping review of the convictions, conventions and developments around quality in qualitative research

**DOI:** 10.1098/rsos.242001

**Published:** 2025-06-25

**Authors:** Xavier Salet, John Gelissen, Guy Moors, Jelte Wicherts

**Affiliations:** ^1^ Department of Methodology and Statistics, Faculty of Social and Behavioral Sciences, Tilburg University, Tilburg, The Netherlands

**Keywords:** scoping review, meta-research, source criticism, transparency, qualitative research, social sciences

## Abstract

We present a scoping review of methodological papers in the social science literature covered in Scopus from 2017 to 2022. In this review, we document the shared norms, ideals and practices regarding the quality of qualitative research methodology. More specifically, we examined the regularly proposed idea that qualitative methodology is so diverse that it is unfeasible to establish shared quality standards. Coding of 111 articles yielded 17 categories that relate to key topics discussed in publications on research quality in qualitative research, such as the quality of the research process, integrity, reflexivity, ethics and transparency. These codes reflect both established ideals and new developments. We conclude that articles in our sample have many shared values in common, but that these values at this point do not yet translate into shared research practices or a common core for quality evaluation.

## 1. Introduction

The question of what constitutes quality can be divisive among qualitative methodologists in social science research. A notable illustration of how highly views on research quality can diverge is an exchange between two prominent qualitative methodologists, Norman Denzin and Martyn Hammersley. Disagreeing with a book [1] in which Hammersley criticized arts-based methods, Denzin published a script for a theatre play titled 'Apocalypse Now: Overcoming Resistances to Qualitative Inquiry' [2]. In the abstract, Denzin states that Hammersley’s criticism is based on misrepresentations and proceeds with a dramatized dialogue between different methodologists, including Hammersley and Denzin themselves, as characters. Hammersley responded with another article: 'Research, Art, or Politics: Which Is It To Be?' [3]. In this article, he makes a case for factual argumentation based on evidence and argues that arts-based elements obscure this [3].

In itself, such a methodological dispute is not uncommon, but their disagreement illuminates such divergent viewpoints on research quality that they touch on the question of what should be seen as academic research to begin with. Hammersley and Denzin’s disagreement reflects a methodological sphere with a wide range of attitudes towards the topic of research quality. Indeed, many scholars have already noted the wide variation around interpretations of the quality of qualitative research [4–6]. High diversity, fragmentation and idiosyncrasy in qualitative methodology are mentioned by both those who welcome it [7,8] and criticize it [4,9,10]. This complicates efforts to identify shared quality norms or reach consensus on standards [11,12], even if this has been a topic of interest in many social science fields [13–17].

Qualitative research methodology thus encompasses a rich array of perspectives and approaches [6]. Yet, despite its breadth, ‘qualitative research’ is prevalent as an overarching term for several methods, ideals, epistemologies and tools that researchers commit themselves to and find like-minded peers in [11]. This is shown in the vast and ever-growing collection of methodological works and journals that address qualitative research as a common frame of reference [12–14]. Thus, the question becomes what unites this group of diverse methodologists who ascribe to the overarching framework of qualitative research, and what grounds exist in the literature for their communal frame of reference?

While qualitative methodologists propose various ideals and principles that bind them, such as a commitment to developing nuanced contextual insights [15], openness to complexity [16] or usage of critical paradigms [17], there is little literature available that broadly investigates these perspectives for their places in the wider contemporary methodological literature. For this reason, it is beneficial to develop insights into what topics of interest receive current importance. To do so, we designed a scoping review of recently published methodological articles on the quality of qualitative research. We aimed to move beyond earlier conclusions that shared standards are hardly feasible and to explore whether the common principles that qualitative methodologists highlight also translate into a common core of practice. We address the following research questions:

(1) Which key topics around methodological quality can be identified in methodological papers about qualitative research in the social sciences?
(2) How do qualitative methodologists translate shared ideals into shared research practices?
(3) Can we identify a unified core for evaluating qualitative research quality?

### 1.1. Contribution to the literature

In this scoping review, we will provide a thematic overview of topics and discussions that receive attention in the contemporary literature and accompany it with a critical evaluation. Furthermore, we aim to further insights by looking both back at how views originated, and ahead to how shared notions of research quality can be envisioned.

In our discussion, we will deepen our understanding of our findings by relating them to seminal debates in the qualitative research literature. We show not only what is currently important in qualitative methodology but also how this relates to canonical developments in methodological thought. As qualitative research receives increasing attention from many disciplines [18], our search for these unifying themes and continuations becomes pertinent.

Although not part of our original coding scheme, we introduce source criticism as an interpretive angle that emerged from reflection on our empirical findings. Source criticism refers to the practice of scrutinizing empirical sources themselves for their quality or adequacy in answering research questions [19]. The focus here is on why a source gives the information it gives [20], what can be concluded based on this [21] and whether researchers report transparently on these matters [22]. By considering source criticism, we aim to provide a shared vantage point for the assessment of research quality that encompasses different perspectives from our sample.

### 1.2. Positionality statement

This research is carried out by a PhD candidate (the first author) and his supervisors. The first author has a background in qualitative sociology. The second and third authors have used qualitative research approaches to evaluate the quality of survey data. Furthermore, the second author has supervised qualitative and mixed-method PhD research projects in the fields of social sciences and law and taught qualitative research methods at the undergraduate and graduate level for several years. As a meta-research group, we are strongly motivated to understand common research practices in the social sciences and to see where there is potential for improvement. For the second, third and fourth authors, the topic of this scoping review was a relatively new field of inquiry, while for the first author, this scoping review was his first project for his PhD. The process of undertaking this scoping review was therefore an educational process for all of us.

## 2. Methodological approach

To get an adequate view of contemporary viewpoints, we investigated articles that were published at most 5 years ago from the start of our project. Because the project began in 2022, we included articles published between 2017 and 2022.^1^ A scoping review was deemed the best strategy, as we aimed to explore shared conceptualizations of research quality through close reading of research articles. This largely overlaps with Munn and colleagues’ description of a scoping review as a method ‘to identify and map the available evidence’ [23], and as a good fit for ‘[the clarification of] key concepts/definitions in the literature’ [23]. This was considered to suit our exploratory purpose better than another format, such as a systematic review that typically evaluates the evidence on more specific topics [23].

In its search function, Scopus makes use of ‘Subject Area Classifications’ (for an updated overview of Subject Area Classifications, see [24]). We specifically only used articles within the ‘Social Sciences’ subject area classification and excluded areas such as ‘Psychology’ or ‘Nursing’. We did this because ‘Social Sciences’ is a broad category that, within Scopus’ infrastructure, does not place an exclusive focus on any (sub)discipline. Including the Subject Area Classifications ‘Psychology’ and ‘Nursing’ would have caused over-emphasis on these categories. ‘Social Sciences’ does not completely exclude articles that fall under these other categories, as we still found such articles in the final sample. However, it left more room for articles from other disciplines that Scopus did not assign a major Subject Area Classification, such as sociology, anthropology, political sciences, organizational studies and social work.

We also excluded the Subject Area Classification ‘Arts and Humanities’ from our search. This exclusion is because social sciences over the twentieth century came to have a specific place in academic research, defined by a relatively applied focus on contemporary social organizational issues [25–28]. So, while research methods overlap between the humanities and social sciences, qualitative research methodology can be seen to have a distinct development in both fields [29]. Because of this, disciplines strongly related to the humanities, such as history, literary sciences or cultural studies, are largely absent from our sample.

**Figure 1.**
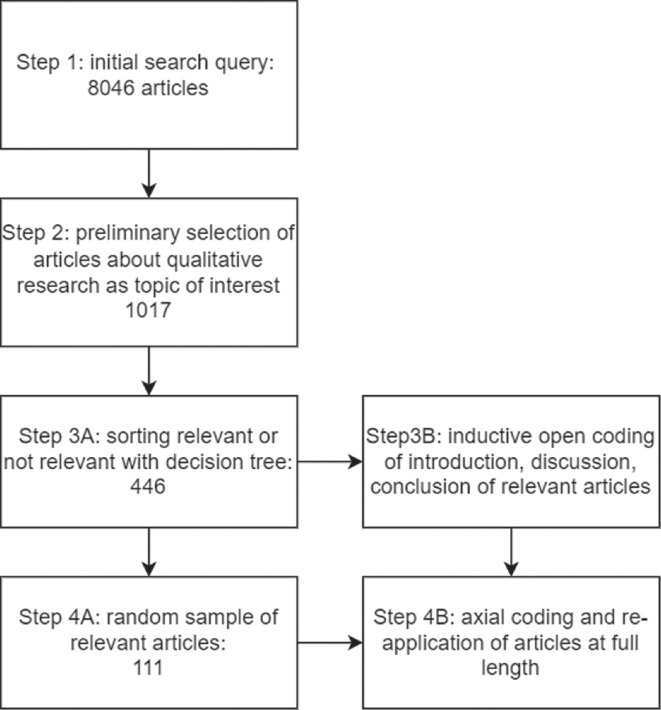
Overview of selection procedure.

As our review is explorative, we developed a research protocol that draws from different research approaches, combining a systematic search procedure with deep reading in the analysis phase. For the selection of articles, we developed a search query and a selection procedure to be carried out by research assistants. For the content analysis, we opted for a more flexible approach and worked from inductive open line-by-line coding towards axial coding. The specific steps taken are shown in figure 1 in the next section.

### 2.1. Overview of the methodological process

The selection of articles involved several steps—details of each step are provided in the electronic supplementary material, appendix A. We started with a search query in Scopus (search queries are given in electronic supplementary material, appendix A), from which 8046 articles emerged (step 1). In step 2, we made a broad preliminary selection of articles on methodology with the help of the AI tool ASReview [30], resulting in 1017 articles. In step 3A, we opted for purposive sampling by categorizing the 1017 articles into relevant and not relevant using a decision tree—which was applied by five research assistants—and can be found in electronic supplementary material, appendix B. Articles were retained if their abstracts addressed at least one of the criteria listed in table 1. The student assistants reached a consensus on 71% of the articles. For the remaining 29%, the first author made the definitive decision for either inclusion or exclusion.

**Table 1.** First step in the decision tree of step 3A.

is one of the following aspects present in the abstract?	definition
primary qualitative research	research that directly employs empirical qualitative research and does not employ quantitative methods
process quality assurance	efforts towards or discussions about shared measures or procedures to check or guarantee the different phases in the operational process are done in a thorough and trustworthy way
transparency	the availability of primary research data and insight into how this was obtained; clarity about the procedures, methods, technical communication with participants and fieldwork materials that were used by the outside world
integrity	the principle that research is done in an honest manner, and that the researcher is faithful to basic principles of academic research (researcher impact on data)
reflexivity	critical analysis of how the specific positionality of the researcher and/or their institutional power influences the research process
ethics	codes of conduct for engagement with participants to avoid doing harm (prescribed); decisions made during the research and their effects on participants (operational)

We arrived at these conditions after several test runs on the original data set. We found that the topics under these conditions together captured most articles that deal specifically with operational questions of qualitative research—as opposed to, for example, theoretical or philosophical questions. With the selection of these conditions, we aimed to keep a broad base of articles that leave leeway to include articles that interpret these terms in specific ways.

### 2.2. Analysis

Our purposive selection process yielded 446 relevant articles as the basis for analysis. These are articles that were identified in step 2 as being about qualitative research methodology and subsequently selected as relevant in step 3A. We applied inductive coding to the 446 articles selected in step 3A. As this set was large, we coded only the introductions, discussions and conclusions. As the dataset proved too large to be explored in depth, in step 4A, we took a random sample of 111 articles. This amounts to 25% of 446 and thus was deemed enough to capture an accurate representation of all the different topics and viewpoints present in the total sample. The reference list for these 111 articles can be found in electronic supplementary material, appendix E. The open codes from step 3B were grouped into higher-level categories (referred to as ‘category codes’ in Atlas TI), which we re-applied to the full text of the final sample of 111 articles and refined during application to the full texts of the 111 sampled articles.

**Figure 2.**
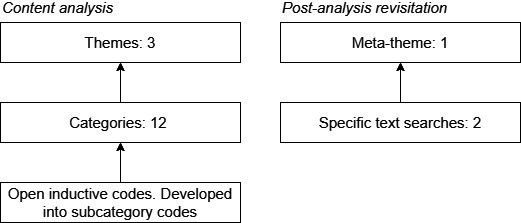
Overview of the hierarchy of codes and themes

In total, the open codes amounted to 15 larger categories, of which 12 were put under larger themes. After this, three categories remained that we decided to exclude from our analysis for having little analytical value. We also developed one meta-theme—a theme not derived from the conventional empirical procedure, but from post hoc reflection on our results and focused reassessment of our sample. This meta-theme, *source criticism*, provides a comprehensive perspective on research quality that could unify articles written from different vantage points on research quality. An overview of the themes and meta-theme can be found in figure 2.

## 3. Results

Out of a total of 15 categories that were developed and applied in steps 3B and 4B, 12 formed the basis for three themes—see table 2 for an overview of all the themes and their categories. Each category has a range of subcategories that entail different viewpoints related to the category. The reference to the specific subcategories can be found in the footnotes. An overview of all categories and their subcategories, including the three categories that we do not use for the themes, can be found in electronic supplementary material, appendix C. This appendix also contains an overview of which (sub)categories were attached to which article from the sample; for a more detailed overview, appendix D contains all the text excerpts that were coded.

**Table 2.** Themes and categories developed in analysis.

theme 1: epistemological characteristics of qualitative research and tensions with standardization	theme 2: researcher obligations and societal impact	theme 3: structural changes and institutional actors
seeking complexity	role of the participant	transparency/open science/data archiving/sharing
high diversity of qualitative approaches	reflexivity	digitalization, online research and algorithms
qualitative (interpretive) research quality standards as opposed to quantitative ((post)positivist) research quality standards	social justice, decolonization and activism	institutional influences
standardization of quality	academic research and society	writing + journal conventions
**meta-theme: source criticism^a^**		

^a^ This meta-theme was derived from post hoc reflection on the themes above and was not part of conventional empirical procedure.

### 3.1. Theme 1: epistemological characteristics of qualitative research and tensions with standardization

The first three categories represent typical epistemological characteristics of qualitative research. In our sample, the image emerges that the fourth category, *standardization of quality,* can be at odds with the characteristics of qualitative research explored in the first three categories.

#### 3.1.1. Seeking complexity

Several aspects that articles in our sample say make qualitative research unique come together under this category.^2^ For example, gaining in-depth knowledge of the complexity and richness of a social phenomenon^3^ and deep immersive engagement with the topic of research^4^ are mentioned. Also important is that qualitative research is discussed as a more implicit undertaking in which tacit knowledge is sought between the lines.^5^ Here, the subjectivity of the researcher is mentioned to shape the research^6^ and qualitative research is seen as creating knowledge in the form of a unique perspective on the world.^7^ This unique perspective is created by focusing on specificities, as small sample sizes are accepted and often valued.^8^ Furthermore, qualitative research can be presented as a methodology that requires flexibility and preparation to deal with unpredictable circumstances.^9^ Two articles in our sample illustrate how these characteristics relate to methodology: one introduces an interview method to reach closer to the bottom of the life worlds of interviewees [31] while another offers a reflection on how coding schemes can reach more depth [32].

It is mentioned that it can be challenging to process the large amounts of information that typically arise from qualitative research:^10^ when is analysis finished, and when have enough perspectives been aggregated? An answer provided in the sample [33] is that it is simply not possible to grasp the full meaning of qualitative research. Because of this, researchers must limit their choices:^11^ ‘[…] I found it initially difficult to fully understand how we could (re)present all that was mentioned in the interviews into a few stories’ [33].

Regarding the role of theory and epistemology/ontology, we observed divergent viewpoints.^12^ Whereas other subcategories have a high consensus on what good qualitative research can achieve, here viewpoints diverge. We found that there appears to be a tension between the empirical nature of qualitative research and the importance of the theoretical framework in providing explanations. As subjectivity and implicitness are well-accepted and well-established elements in qualitative research, the theoretical framework is prominent in both explaining and exploring the phenomenon at hand. As it was phrased in a sampled article, there is a risk for over-theorizing [34]: ‘Do we so often want to see ourselves as “theoreticians” for whom the empirical world is sometimes little more than a springboard for our conceptual work and sometimes a place to be unproblematically plundered for examples that demonstrate the applicability of the theories on which we have already settled?’

#### 3.1.2. High diversity of qualitative approaches

Within this category,^13^ our investigated articles discuss the fact that the methodology has a wide array of approaches and epistemologies.^14^ This surfaces in this example from an editorial that provides general guidelines for qualitative research: ‘Regardless of your rhetorical style—which can range from the near-positivistic to the nearly literary—all [high-quality qualitative research] should incorporate transparent markers that enable readers to follow a coherent and consistent narrative route*’* [35].

The downside that is portrayed in our sample is that in relation to quality assurance, it becomes difficult to agree on shared norms^15^: ‘There is a lack of communication between different camps in qualitative research. A good way to resolve this would be to revisit the concepts and find common ground.’ [36]. A critique in papers mentioning this issue is that quality terms can mean something different depending on a researcher’s epistemological background, or because opinions differ on what constitutes quality altogether. This diversity, or fragmentation, complicates efforts towards standardization—see the category *standardization of quality* further on in this theme for more information.

#### 3.1.3. Qualitative and quantitative research standards

This category is applied to articles that mention the difference between qualitative quality standards and quantitative standards.^16^ Most often, these are put in opposition to one another, where the former represents interpretivism/constructivism, and the latter represents a value-free search for facts.^17^ A subset within this category consists of articles from disciplines dominated by quantitative research methods.^18^ In these disciplines, qualitative researchers introduce qualitative research as a methodology to advance knowledge or sometimes express discontent with the lack of concern for their methodology. This surfaces, for example, in an article that opts for more consideration of qualitative methods and epistemology in the quantitative-dominated discipline of engineering education research [37].

However, we also encountered articles that bridge the gap between qualitative and quantitative.^19^ These are articles that focus on how both objectivist and interpretivist elements can be present in qualitative research: ‘Thus, rather than give up on the notion of objectivity, it is better to promote an understanding of objectivity which may serve as a reachable—and useful—guiding ideal in qualitative research’ [38].

#### 3.1.4. Standardization of quality

The articles in our sample discuss standardization in a broad sense.^20^ It refers to standardization as the development of formal protocols, but it also refers to more loose discussions around the development of shared quality norms or methodological guidelines. This category is one in which there is considerable internal division. On the one hand, articles acknowledge that qualitative research has few universal quality standards,^21^ often has problems properly reporting on methodological quality,^22^ would benefit from standardization^23^ or requires better training.^24^ On the other hand, articles provide criticism on, or discussion about the feasibility of, standardization. For example, articles that express a positive attitude towards standardization often also question its feasibility in qualitative research or argue that standardization should not be too tightly imposed.^25^ The following quotation showcases this ambiguity:

Our findings contribute further to the formalization of justifiable norms in health and social science and aid phenomenology in moving toward Giorgi’s perspective that a method should be clear such that it is useable by any researcher. This is not to say that all phenomenological studies should look the same; rather, that researchers can turn to these findings as a source of greater scientific clarity, especially given psychologists’ concerns that qualitative samples are often 'too small’. [39]

This quotation, from a paper that strongly endorses standardization of qualitative research practices, addresses a fear that qualitative research will become too formalized.

A more specific criticism of standardization is the warning that qualitative research will become a box-checking endeavour for bureaucratic purposes:^26^

Indeed, we hear from researchers who use our *reflexive* [thematic analysis] approach […] but reference data or thematic saturation in their publications, because reviewers and editors *required* it, often citing checklists like COREQ or CASP. And researchers often pragmatically acquiesce to reviewers’ and editors’ demands, even though they hold some critique or question of (data) saturation. For these researchers, the concept of (data) saturation is deployed as the rhetorical device we mentioned earlier, a 'quality assurance' mechanism to get 'passed' by the gatekeepers of knowledge. [40]

### 3.2. Theme 2: researcher obligations and societal impact

The first three categories highlight the moral obligation felt by many qualitative researchers in our sample towards their topics, participants or the communities with which they conduct research. While the first two categories represent ideal attitudes that are quite well-established in qualitative research in general, the third category, *Social justice, decolonization and activism* represents a subbranch of qualitative research with a strong emphasis on emancipation of marginalized communities. In all three categories, a desire for an authentic research account that stays as true as possible to the perspectives of the participants/participants’ communities surfaces. The fourth category, *Academic research and society* refers to how qualitative research is related to and could impact society.

#### 3.2.1. Role of the participant

Articles within this category generally advocate for enlarging the influence of the participant on the research outcomes.^27^ Notably, half of the articles within this category explicitly discuss power relations between participant and researcher.^28^ Here, the active involvement of participants is central to the research process.^29^ In participatory research or member checking, for example, participants are given a prominent role in research planning, data gathering or analysis. Among articles in our sample that discuss these topics, there is consensus about the need for participant emancipation. This is illustrated in the following article that introduces an immersive method to empower participants:

In this article, we aim to introduce [digital storytelling] as part of a feminist strategy in research methods allowing women to tell their stories in their own words through a guided creative process that includes co-production of personal stories and—in so doing—further addresses the issue of power imbalance in the research process*.* [41]

Similarly, articles present qualitative research as a process of co-creation of knowledge between researcher and participant.^30^

Another way in which the strong commitment to participants surfaces in the articles is through addressing issues related to participant wellbeing. Many of the articles under this category discuss how to deal with participant vulnerability, the issue of participant harm or researching sensitive/controversial topics.^31^ A final topic that is discussed much, albeit not necessarily in an emancipatory manner, is the relationship with the participant, (informed) consent and the establishment of trust between researcher and participant.^32^

#### 3.2.2. Reflexivity

Reflexivity is a highly valued quality aspect in the methodological articles.^33^ In discussions about quality maintenance, reflexivity often surfaces as an important check. Researchers are expected to think about their own identity, background and ideals. This can be done to check biases,^34^ but also as an extension of transparency.^35^ Researchers are reflexive in research communication to provide insight into how they got to their conclusions and what drove them in the research process.

Reflexivity can also be implemented in a more pragmatic way, namely to investigate the positionality of the researchers in relation to their subjects or participants.^36^ Here, researchers create awareness of how they relate to the group they are researching or the people they are talking to and establish which aspects make them insiders and which aspects make them outsiders. Subsequently, research planning will be adjusted to this. Positionality is not necessarily either inside or outside, but complex, fluid and depending on the specific situation of the research, as is shown in the following quote from our sample:

For example, although I am an African American woman, I did not assume that I was granted immediate access to interview women from this group based on my ethnic background. Women who were in this exclusive group of the study were considered wives of the incarcerated; therefore, I was still considered an outsider. [42]

#### 3.2.3. Social justice, decolonization and activism

Articles within this category^37^ often have a strong motivation to give voice to marginalized communities or to empower their research participants^38^: ‘This article reflects on a research project, which was conceived as participatory in the sense that we aimed to give voice to a hitherto marginalised and silenced group in society, namely children and their families who had been the subjects of a child protection investigation […]’ [43]. Many articles relate these emancipatory goals to efforts of attaining real-world impact.^39^ In this way, activist research can be seen as a mix between the academic endeavour to search for and curate knowledge, and the perceived interests of participants or their community.

Another topic that is discussed in articles within this category is that of decolonization, both in general and in academic research.^40^ Among these articles, the problematic history of colonization is mentioned and the hegemony of the Western world is problematized. In this way, qualitative methods aid researchers in their attempts to redistribute power in shaping public discourse: *‘*Undoing duality and removing the othering creates an important opportunity to move beyond exclusively colonized knowledge […], an approach that is consistent with the Interpretive Description approach’ [44].

#### 3.2.4. Academic research and society

Several articles within this category^41^ discuss balancing the interests of the researcher, participants, universities, funders or other institutional actors.^42^ Furthermore, some articles focus on returning value to participants or enhancing the practical use of research beyond academia.^43^ This attitude is illustrated well by the following article that presents its method as both emancipating and therapeutic: ‘Attempting to rectify the condition of being silenced by rationalizing voice-giving to the “voiceless”, body-mapping literature has documented its therapeutic, pedagogical, artistic, communicative, political and investigative potential as both a method and therapy […]’ [45].

### 3.3. Theme 3: structural changes and institutional actors

The first two categories describe emergent elements of qualitative research that are likely to alter how qualitative research is done. The third category, ‘Institutional influences’ focuses on which institutions influence the conduct of qualitative research, and which institutions are perceived to be involved in developing the newly emergent quality elements described in the first three categories.

#### 3.3.1. Transparency/open science/data archiving/sharing

We found that sampled articles generally portray transparency positively. It is commonly seen as a fundamental aspect of proper research conduct, that is important in maintaining the trustworthiness of qualitative research.^44^ A segment of articles defines transparency in relation to participants, focusing on how they are represented in research output and the challenges that arise here.^45^ Several articles problematise a lack of transparency or refer to the vague character of qualitative reporting.^46^

Several emergent topics indicate a progression towards new forms of transparency: data sharing/archiving,^47^ secondary data analysis,^48^ data curation/preservation,^49^ auditing qualitative research^50^ or open science.^51^ Related to these developments are articles that discuss questions of data ownership or power relations in the data curation process.^52^

It is therefore worth illuminating two perspectives from our sample on data reuse and its potential benefits for respectively empirical understanding and the wellbeing of participants: Dodds *et al.* synthesized 12 different qualitative datasets spanning 16 years about the experiences of HIV patients:

We wanted to see if it was possible to assemble a large set of qualitative UK social science datasets for re-analysis, enabling a deeper exploration of the changing nature of engagements with ARVs over time. […] We surmised that this process would help to develop rich insights, rather than collecting even more new data in the here-and-now. [46]

In an article that explores the other side of the process, namely archiving interview data, Stewart and Shaffer give the following argument: ‘Interviews with refugees can be long and emotionally stressful but having invested their time and energy, it would seem to be beneficial to utilise the data for multiple projects’ [47].

#### 3.3.2. Digitalization, online research and algorithms

Articles within this category focus on the influence of digitalization on qualitative research.^53^ Roughly, articles deal with single online methods^54^—e.g. online interviewing—or describe the impact of single websites, communication channels, archives or databases on qualitative research.^55^ All these are owned and maintained by different entities with their own policies. As the following quote illustrates, this complicates the research process:

*Second, access requires commitment. If researchers treat leaked material as an archive like any other, they are accountable for doing it justice, which invariably requires more than a citation or two. Even small claims, backed by primary sources, require scrutinizing those documents’ original communicative context and assessing biases in source repositories and in researchers’ sampling and interpretation of particular texts, as well as ethical concerns. With the proliferation of online sources, ease of access should not imply ease of analysis* [48].

This underlines the increasing complexity of online information infrastructures. Other discussions are about the ethics of online research^56^ or the impact of COVID-19^57^—we note that this last point is significantly mitigated due to our decision in step 3A to exclude articles that specifically focus on the impact of COVID-19 on qualitative research.

#### 3.3.3. Institutional influences

This category was identified when articles discussed the larger institutions that are involved in qualitative research.^58^ These are mainly ethics review boards^59^ and funding bodies.^60^ Regarding the latter, articles ascribe the increasing influence of institutions on defining and imposing certain practices. For example, it is mentioned that funding bodies increasingly demand data archiving.^61^ Another example is that articles mention that funders cause time pressure.^62^

Several articles mention the interaction between qualitative researchers and ethics review boards.^63^ Specifically, a discrepancy between the values of the review boards and those of researchers arises in our sample: ‘However, as a form of procedural ethics, institutional ethics review has its critics […]. Such upfront processes may potentially impact researchers’ rights […] and constrain the research process […]’ [49].

#### 3.3.4. Writing and journal conventions

We applied this category to articles discussing not the research itself, but the processes involved with communicating qualitative research.^64^ Some topics that arose in our sample is criticism on the pressures in academic publishing^65^: *‘*Too often there is an emphasis on quantity (at the expense of higher quality research), especially in certain countries where there is an emphasis on "publication numbers" when applying for job or tenure […]’ [33]. Another point mentioned in relation to writing and journals is the limited writing space for qualitative research in journals.^66^

### 3.4. Meta-theme: source criticism

We developed this theme after we finished our analysis of the previous three themes. In reflecting on our results, we found that many of our categories foreground diversity or idiosyncrasy. Furthermore, many quality ideals that we describe have a high level of abstraction—analytic, intellectual or theoretical engagement with data after collection—or about ethical and moral responsibilities. While it might be an easy task to reach an agreement *that* these topics are important, they do not readily facilitate a straightforward discussion on shared notions of good or bad research quality. An example of this is participant involvement or feedback.^67^ These discussions mainly are about involving participants in analysis or having them give feedback on the interpretations of researchers. This practice is highly focused on the subjective realities of the researcher and participant or on the researcher–participant rapport, and is therefore difficult to standardise.

Based on this observation, we reflected on whether we overlooked aspects that might provide grounds for common standards of empirical conduct that could bridge the various viewpoints that we encountered in our sample. While re-assessing the codes and themes that we developed, we noted a practice that few of our categories and themes explored. This is source criticism, or the practice of assessing sources themselves for their quality or adequacy in answering research questions [19]. Questions related to this are why a source gives the information it gives [20], what can be concluded based on the source [21], and transparent reporting about this [22].

As our initial research questions revolved around broad exploration, we were interested to see if, with more specific search queries, we could find instances of source criticism. For this, we used a twofold approach. We first looked whether there were subcategories in our original coding framework that could lead us to instances of source criticism. We found two subcategories that deal with a more critical outlook on the participant, which are *Disagreement between researcher and participant*^68^ and *Participant recruitment.*^69^ We found one that could *Sampling and sample size*,^70^ a subcategory of *Scale and big data*. For the second step in our approach, we made a new search query in Atlas TI. This is because our research is exploratory, meaning that the absence of source criticism could also be a matter of our focus on coding. This search query sought for the combination of ‘source’/‘document’/‘participant’/‘interview’ AND scrutin*/quality, legit*/assess*/trustworthiness/valid*/critic*/credib*/ and authentic*. Through revisioning the aforementioned three subcategories and by sifting through all the paragraphs that the search query presented, we found 10 articles that touch upon the topic of source criticism.^71^

Two articles stood out to us because they devote their main arguments to the topic of source criticism. What is particularly relevant for our review is that these represent quite different spectrums of the qualitative research realm: one article focuses on social justice-oriented qualitative research with marginalized groups [49], the other focuses on the trustworthiness of WikiLeaks documents in political sciences [48]. Motivated to help participants from ethnic minorities, the former article [50] describes how researchers run into dilemmas when their participants’ insights do not seem trustworthy. This concerns the fact that participants might downplay or withhold information about their experiences in a racist system that marginalises them: *‘*Interview narratives are all too often accepted as an authentic, true voice, representing experience without an analysis of what is being represented and for what purpose*’* [50]. The article further explains how the researchers dealt with this problem. The latter article [48] focuses on the usage of WikiLeaks documents as evidence in political science research and the pitfalls that come with uncritical assessment of how these documents were produced.

It should be noted that source criticism goes beyond mere sampling procedures, gaining access or member checking, topics we found regularly across our sampled articles and that surface under other themes. What stands out among the articles that we found, is that they question aspects of their sources themselves, which could, for example, be about potential deception by participants [38] or the intentions behind people’s participation in research [43]. The article that we highlighted by Maiter & Joseph [50] shows that this does not have to be about the dismissal of participants as valuable sources, but could also be a motivation to take extra care to facilitate them in sharing their insights.

## 4. Discussion

In our scoping review, we investigated a sample of 111 academic articles that focus on qualitative research methodology. In this discussion, we will answer the three research questions with the insights that we derived from the review. We will also contextualize our insights with wider academic literature on qualitative research methodology. Our research questions stated at the start of this article were as follows:

(1) Which key topics around methodological quality can be identified in methodological papers about qualitative research in the social sciences?
(2) How do qualitative methodologists translate shared ideals into shared research practices?
(3) Can we identify a unified core for evaluating qualitative research quality?

The three themes that were identified in our empirical analysis—(1) *epistemological characteristics of qualitative research and tensions with standardization*, (2) *researcher obligations and societal impact*, and (3) *structural changes and institutional actors*—all provide answers to research question 1. These three themes are often discussed in our sample and are clearly linked to the wider methodological literature.

In Theme 1*—epistemological characteristics of qualitative research and tensions with standardization*—an important finding was that high regard for complexity and diversity of approaches could stand in tension with academic demands for standardization. Furthermore, the uneasy relationship with quantitative research was highlighted here. These topics go back towards the early formalization of qualitative methods and can be found in several methodological works since.

For practical examples, early pioneers in the formalization of urban sociological methods and anthropological methods provide insights: the Atlanta School of sociologist William Du Bois, the research group centred around anthropologist Bronislaw Malinowski at LSE in London and the Chicago School of Sociology, all having their heydays in the late nineteenth century and/or first half of the twentieth century. William Du Bois and the Atlanta School in the late nineteenth and early twentieth century refuted widely accepted racist views through pioneering meticulous ethnographic research and transparent methodological reporting on the living conditions of African Americans [51–53]. Malinowski, having a background in physics, developed standardized forms for his researchers in which they would have to fill out observations under each category (e.g. economics, social, organization and language [54]). In these efforts, and parallel with many articles in our sample, Malinowski ran into the limitations of standardization as well: his forms never became a universal standard, and many of his researchers struggled with adjusting their insights to the mould that the form provided [54]. Furthermore, apart from these early systemizing attempts, Malinowski’s group also pioneered interpretivism. They did this by using personal relationships and rapport as a legitimate form of researching the intricate meaning-making processes of the societies they investigated [55]. The examples of Du Bois and Malinowski show that standardization aids qualitative research quality, but that it can also be at odds with the goal of gaining deep interpretive understanding. The articles in our sample convey that finding a midway between these two is still a major challenge in establishing quality norms.

Our sample’s duality between empirical standardization and local contextualization also reflects larger debates that emerged in the development of social theoretical thought. For example, in nineteenth-century sociology, Auguste Comte [56] and subsequently Émile Durkheim [57] envisioned social research as finding the structural workings of societies in an objective fashion [58]. On the other hand, sociologist Max Weber emphasized the role the individual researcher plays in interpreting social phenomena [59].

Related to the discussion on standardization versus interpretation is the qualitative/quantitative divide that we found in our sample. Here, twentieth-century developments again provide context to understand how viewpoints in our sample were shaped. More specifically, they were the relatively negative attitude towards quantitative research originated. At the University of Chicago, debates between proponents of contextual case study methods on one side and of structurally abstractive statistical methods on the other got heated after the Department of Sociology started expanding its work on statistical methods in the 1920s [60]. This dispute can be seen as a prelude to what would grow into the qualitative/quantitative divide that emerged in the 1950s and 1960s [14], which eventually became so big that it is often referred to in qualitative methodological literature as the ‘paradigm wars’ [61–64]. The qualitative side would become influenced by constructivist and interpretive methods such as symbolic interactionism [65] or ethnomethodology [14], while the quantitative side would move on to develop statistical methods further.

The continuation of these longstanding debates in our sample highlights their deep roots in the methodological literature. Especially regarding the discussions around quantitative research, remnants of the harshness of the earlier days can still be found in our sample. For some, *not* being a quantitative researcher can be a relevant aspect of being a qualitative researcher. This indicates that, despite efforts at integrating qualitative and quantitative research—e.g. mixed methods research [66]—there is still a relevant divide between quantitative and qualitative researchers, or at least that there are qualitative researchers who experience this divide strongly.

Regarding Theme 2*—researcher obligations and societal impact*—the categories that we found around participant wellbeing, reflexivity and social impact/activism are more rooted in the developments following the 1960s. This is when the term ‘qualitative research’ became institutionalized. An example of what grew out of this development is the book 'Naturalistic Inquiry' by Lincoln and Guba that was published in 1985 [67]. This book, now a standard in qualitative methodology, introduced the epistemological premises of social constructivism and interpretivism to broader audiences. At the same time, researchers began questioning the problematic history of social research methods, the suppressive colonial practices in which it had been engaged, and an all too easy acceptance of the idea of science as objective [68]. As philosophies such as feminism and critical theory gained increasing attention, a more ethical stance towards participants [68] and an emphasis on researcher reflexivity became important aspects of qualitative research [69]. The social scientific disciplines also received internal critique for their facilitating role in, for example, American expansionism [70]. During this time, qualitative research became increasingly politicized, with the discipline becoming home to those who wanted to challenge the status quo or help marginalized people [71]. A great commitment to the abovementioned topics can be found in many articles in our sample, which indicates their lasting influence on the field of qualitative methodology as sources of shared commitment.

Theme 3—*structural changes and institutional actors*—is also historically rooted, as questions about institutional dependency or transparency are timeless. For example, many late nineteenth and early twentieth-century anthropological researchers heavily relied on the infrastructure and authority of the British Empire to do their work [55]. Yet, it is worth highlighting that the discussions that surface in our sample around transparency and increased demands from journals and funders touch upon relatively recent developments. Debates around transparency, openness and data reuse in qualitative research took off in the 1990s. This is illustrated by increased mandatory data sharing standards and the foundation of qualitative data archives since this time [72–77]. The topic further gained relevance in the 2010s after a series of incidents relating to research fraud [78] and replication issues [79] in the field of quantitative psychology. In the years following these incidents, calls for increased transparency in social sciences grew and came to incorporate qualitative research [80–82].

The topics discussed under theme 3 are thus well connected to more current debates in qualitative research around the implementation of transparency, open science and data sharing. Currently, academic discussions on this topic are in full swing. For example, there is a growing body of literature that reflects on the viewpoints of participants [82–88], researchers [75,81,89–94], as well as institutional actors [75,95,96] regarding qualitative data sharing. Furthermore, several scholars are working to introduce qualitative viewpoints into the field of open science [15,74,97–100], which, as discussed in more detail in the previous paragraph, is rooted in quantitative methodology.

Concerning our second research question—*how do qualitative methodologists translate shared ideals into shared research practices?—*we conclude that we could certainly find shared ideals, but they do not translate into shared research practices. We witnessed in our sample that qualitative researchers often aspire towards treating and analysing research topics in original, personalized or creative ways. Furthermore, we see how a strong desire to give voice to participants motivates qualitative researchers to develop methods that fit local contexts and empower participants as active knowledge creators. Transparency and reflexivity are also recurring topics that are emphasized as essential in the research process. However, articles in our sample leave much room for personal interpretation when it comes to putting ideals into practice. This shows, for example, the fact that we could not find concrete examples of widely adopted standardized quality control mechanisms or clear journal guidelines. We thus conclude that qualitative methodologists in our sample generally prefer to give guiding advice, rather than to develop stringent standards. This is a standpoint that is also commonly expressed in the wider methodological literature [101–104].

This reluctance to go further than giving guiding advice also provides the basis for our answer to research question 3—*can we identify a unified core for evaluating qualitative research quality?* Given the reluctance in our sample to work towards shared overarching notions of research quality, we must conclude that we can find a clear core when it concerns broader principles, but not when it comes to evaluating practices.

While we were not able to find a common core of research evaluation in our main empirical search, we do think that our meta-theme, *source criticism* could provide a starting point. Despite it being more in the background in our sample, source criticism does fit into a long tradition in the qualitative methodological literature. For example, in one of the earlier social scientific methodology books, 'Field Studies in Sociology: A Student’s Manual', Palmer states the following:

In the physical sciences much of the technique is crystalized, standardized and transmitted by the use of apparatus. This is, of course, impossible in most phases of sociological research. For this reason social research workers are under obligation to make an especial effort to record their experiences in techniques and to make them generally available*.* [105]

Furthermore, in 'Naturalistic Inquiry', Lincoln & Guba devote much space to emphasizing what they call *auditability—*‘the keeping of records in such a way […] that an auditor can later connect assertions in the report with the raw data on which they are presumably based*’* [67]—in meeting the four trustworthiness criteria of credibility, transferability, confirmability and dependability.

Yet, the literature also points to problems in qualitative research in relation to source criticism, problems that resemble the ones that are alluded to in our sample. Criticism has been expressed on a too-easy acceptance of what surfaces in one’s own source material [106–108], usage of verbal accounts as behavioural proxies [21], unclarity in justifying the inclusion of sources in support of arguments [109] and inaccuracy in referencing empirical data [60,110–113]. A recent illustrative case of these issues is that of the ethnography 'On the Run: Fugitive Life in an American City' [114]. It was initially praised by the sociological community [115] but soon received widespread criticism, for example, around bad referencing of factual statements or an uncritical attitude towards informants’ stories [116–120]. In defence of the publication, scholars mentioned the unstructured or messy nature of ethnography [121,122], and that the critiques the publication received are based on objectivism that is unapplicable to the interpretive and reflexive nature of qualitative research [123–126].

We think that the concept of source criticism has the potential to unite the principles of empirical standardization with those of interpretivism. Looking beyond our sample, we found promising initiatives in this regard, mainly in the field of political sciences. An example is that of Annotation for Active Transparency (ATI). This is a system that allows scholars to link digital annotations in which they elaborate on why they highlight a source, or on other factors that contextualize the source that is used [127]. The usage of ATI was integrated into the Qualitative Data Depository of Syracuse University [128]. It accommodates the interpretive nature of qualitative research. It allows for deep reflexive engagement while challenging researchers to provide insights into how their views come about and how they construct their arguments. Initiatives like these could help qualitative methodology improve its quality and rigour in a way that respects the epistemological premises of the various methodologies that fall under its umbrella.

### 4.1. Future research

As there have been many developments in open science, transparency and data sharing, integrating these practices into qualitative methodology becomes a topic of interest. For example, data archiving is increasingly done by qualitative researchers. Here, it becomes relevant to examine if studies for which the data is archived are also of higher quality. The topic of source criticism could be of guidance in this. If empirical data is more transparently available, this would also allow researchers to more openly engage with it in their argumentation and make transparent why they choose to highlight certain (aspects of) source material. In combination with innovations like ATI, data transparency could thus help researchers improve the quality of their insights. Future research could examine if and how these improvements play out, and best practices could be formulated based on this.

There is also potential for future research into mixed methods methodology and its impact on developing rigorous and useful qualitative methods. In our discussion, we referred to the promise of mixed-methods research. To limit our scope, we left mixed methods research outside our sample, as it was our goal to gain insights into the views that are expressed specifically in qualitative methodological works. We think both qualitative and quantitative methodology would benefit from research into integrating the two.

Finally, we think there is also potential in exploring data sharing as a means to reduce participant burden. One article in our sample went into this question, and we provided additional literature on this topic in our discussion [82–88]. Most participants in these studies are open to the prospect of helping others by sharing their data. As qualitative researchers greatly value the wellbeing of participants, we think there is much potential in exploring how participants’ data can be shared optimally, and how participants can be involved in the curation process.

### 4.2. Limitations

The most notable limitation of this review is that our sample consists of a very broad range of disciplines, each with its own priorities for assessing research quality. Furthermore, academic subfields in our sample define themselves to such detailed extents that it was difficult to make a cutoff. Topics in our sample ranged from dementia care, to political sciences, to sports sciences and everything in between. This broadness made it a challenging task to go entirely into depth.

Only the first author was involved in coding and analysis. This can be considered a limitation, as his observations were not checked. Furthermore, while some (sub)codes were straightforward to assign, such as open science, others were more implicit and difficult to explicate, such as reflexivity, or complexity. Because of this, we want to re-emphasize, as earlier discussed in the methodology section, that our coding here should not be seen as an objective mechanism, but as a tool aiding the first author in exploring the content in depth.

Another limitation is the somewhat low consensus of 71% among research assistants during the sorting of relevant and non-relevant articles in step 3A. While, as established in the method section, this caused an overidentification of relevant articles, this still limited the study in selecting its core desired articles.

## 5. Conclusion

We conducted a scoping review coding 111 methodological articles in the social sciences to explore shared notions of quality in contemporary qualitative research as they appear in methodological articles. Through a content analysis, we provided an overview of certain ideals, as well as current developments and factors that form challenges for qualitative methodology. This resulted in three themes: (1) *epistemological characteristics of qualitative research and tensions with standardization*, (2) *researcher obligations and societal impact*, and (3) *structural changes and institutional actors*. After empirical analysis, we also developed a fourth meta-theme: *source criticism*.

The first three themes provide insights into firmly established discussions in the qualitative methodological community and will sound familiar to those with experience in the methodological field. Our main finding here is that qualitative methodology balances the need for detailed contextual, interpretive and localized accounts with the need for standardized transparent empirical frameworks in which knowledge can be organized and uniformly discussed. In our discussion, we show how these findings relate to the broader literature on qualitative methodology and to historical examples.

While the three main themes represent topics that we found were discussed much in our sample, the meta-theme, *source criticism*, we found to be of less prominence. Therefore, we think there are opportunities for the field of qualitative methodology to expand this discussion. Especially in relation to recent surging attention for transparency, data sharing and re-use, the concept of source criticism can have value. This is because, aside from opening data (where possible), it also aims to make discussions about the value of data more transparent.

## Data Availability

Electronic supplementary material is accessible from OSF [129].

## Footnotes

^1^
For more information on our selection process, see electronic supplementary material, appendices A and B on OSF.

^2^
Subcodes 1A–1T in electronic supplementary material, appendix C.

^3^
Subcodes 1K in electronic supplementary material, appendix C.

^4^
Subcodes 1C and 1E in electronic supplementary material, appendix C.

^5^
Subcode 1P in electronic supplementary material, appendix C.

^6^
Subcode 1O in electronic supplementary material, appendix C.

^7^
Subcode 1J in electronic supplementary material, appendix C.

^8^
Subcode 1H in electronic supplementary material, appendix C.

^9^
Subcode 1F+1R in electronic supplementary material, appendix C.

^10^
Subcode 1G in electronic supplementary material, appendix C.

^11^
Subcode 1B in electronic supplementary material, appendix C.

^12^
Subcodes 1N and 1S in electronic supplementary material, appendix C.

^13^
Subcodes 2A–2G in electronic supplementary material, appendix C.

^14^
Subcode 2D in electronic supplementary material, appendix C.

^15^
Subcode 2A in electronic supplementary material, appendix C.

^16^
Subcodes 3A–3J in electronic supplementary material, appendix C.

^17^
Subcode 3F in electronic supplmentary material, appendix C.

^18^
Subcodes 3E and 3G in electronic supplementary material, appendix C.

^19^
Subcode 3B in electronic supplementary material, appendix C.

^20^
Subcodes 4A–4S in electronic supplementary material, appendix C.

^21^
Subcode 4P in electronic supplementary material, appendix C.

^22^
Subcode 4G in electronic supplementary material, appendix C.

^23^
Subcodes 4B, 4C and 4S in electronic supplementary material, appendix C.

^24^
Subcode 4N in electronic supplementary material, appendix C.

^25^
Overlap between subcodes 4B/4C/4S/ and subcodes 4D/4I/4L in electronic supplementary material, appendix C.

^26^
Subcode 4E in electronic supplementary material, appendix C.

^27^
Subcodes 5A–5W in electronic supplementary material, appendix C.

^28^
Subcodes, 5D, 5J, 5O and 5Q in electronic supplementary material, appendix C.

^29^
Subcodes 5J and 5O in electronic supplementary material, appendix C.

^30^
Subcode 5A in electronic supplementary material, appendix C.

^31^
Subcodes 5B, 5H and 5W in electronic supplementary material, appendix C.

^32^
Subcodes 5S, 5T and 5V in electronic supplementary material, appendix C.

^33^
Subcodes 6A–6E in electronic supplementary material, appendix C.

^34^
Subcode 6B in electronic supplementary material, appendix C.

^35^
Subcode 6E in electronic supplementary material, appendix C.

^36^
Subcode 6A in electronic supplementary material, appendix C.

^37^
Subcodes 7A–7H in electronic supplementary material, appendix C.

^38^
Subcodes 7B and 7C in electronic supplementary material, appendix C.

^39^
Subcodes 7F and 7G in electronic supplementary material, appendix C.

^40^
Subcode 7A in electronic supplementary material, appendix C.

^41^
Subcodes 8A–8H in electronic supplementary material, appendix C.

^42^
Subcode 8F in electronic supplementary material, appendix C.

^43^
Subcode 8H in electronic supplementary material, appendix C.

^44^
Subcodes 9A–9W in electronic supplementary material, appendix C.

^45^
Subcode 9U in electronic supplementary material, appendix C.

^46^
Subcodes 9Q and 9S in electronic supplementary material, appendix C.

^47^
Subcode 9I in electronic supplementary material, appendix C.

^48^
Subcode 9G in electronic supplementary material, appendix C.

^49^
Subcode 9E in electronic supplementary material, appendix C.

^50^
Subcode 9C in electronic supplementary material, appendix C.

^51^
Subcode 9M in electronic supplementary material, appendix C.

^52^
Subcodes 9K and 9O in electronic supplementary material, appendix C.

^53^
Subcodes 10A–10J in electronic supplementary material, appendix C.

^54^
Subcode 10H in electronic supplementary material, appendix C.

^55^
Subcode 10F in electronic supplementary material, appendix C.

^56^
Subcode 10E in electronic supplementary material, appendix C.

^57^
Subcode 10D in electronic supplementary material, appendix C.

^58^
Subcodes 11A–11P in electronic supplementary material, appendix C.

^59^
Subcode 11E in electronic supplementary material, appendix C.

^60^
Subcode 11F in electronic supplementary material, appendix C.

^61^
Subcode 11O in electronic supplementary material, appendix C.

^62^
Subcode 11P in electronic supplementary material, appendix C.

^63^
Subcode 11E in electronic supplementary material, appendix C.

^64^
Subcodes 13A–13I in electronic supplementary material, appendix C.

^65^
Subcode 13F in electronic supplementary material, appendix C.

^66^
Subcode 13E in electronic supplementary material, appendix C.

^67^
Subcodes 5J, 5M and 5O in electronic supplementary material, appendix C.

^68^
Subcode 5C in electronic supplementary material, appendix C.

^69^
Subcode 5N in electronic supplmentary material, appendix C.

^70^
Subcode 12F in electronic supplementary material, appendix C.

^71^
Subcodes 16A-16E in electronic supplementary material, appendix C.
